# Supplementary figures and images for: MMS22L-TONSL functions in sister chromatid cohesion in a pathway parallel to DSCC1-RFC
Source: Life Sci Alliance. 2022 Dec 8;6(2):e202201596. doi: 10.26508/lsa.202201596 (PMC9733570; doi:10.26508/lsa.202201596)

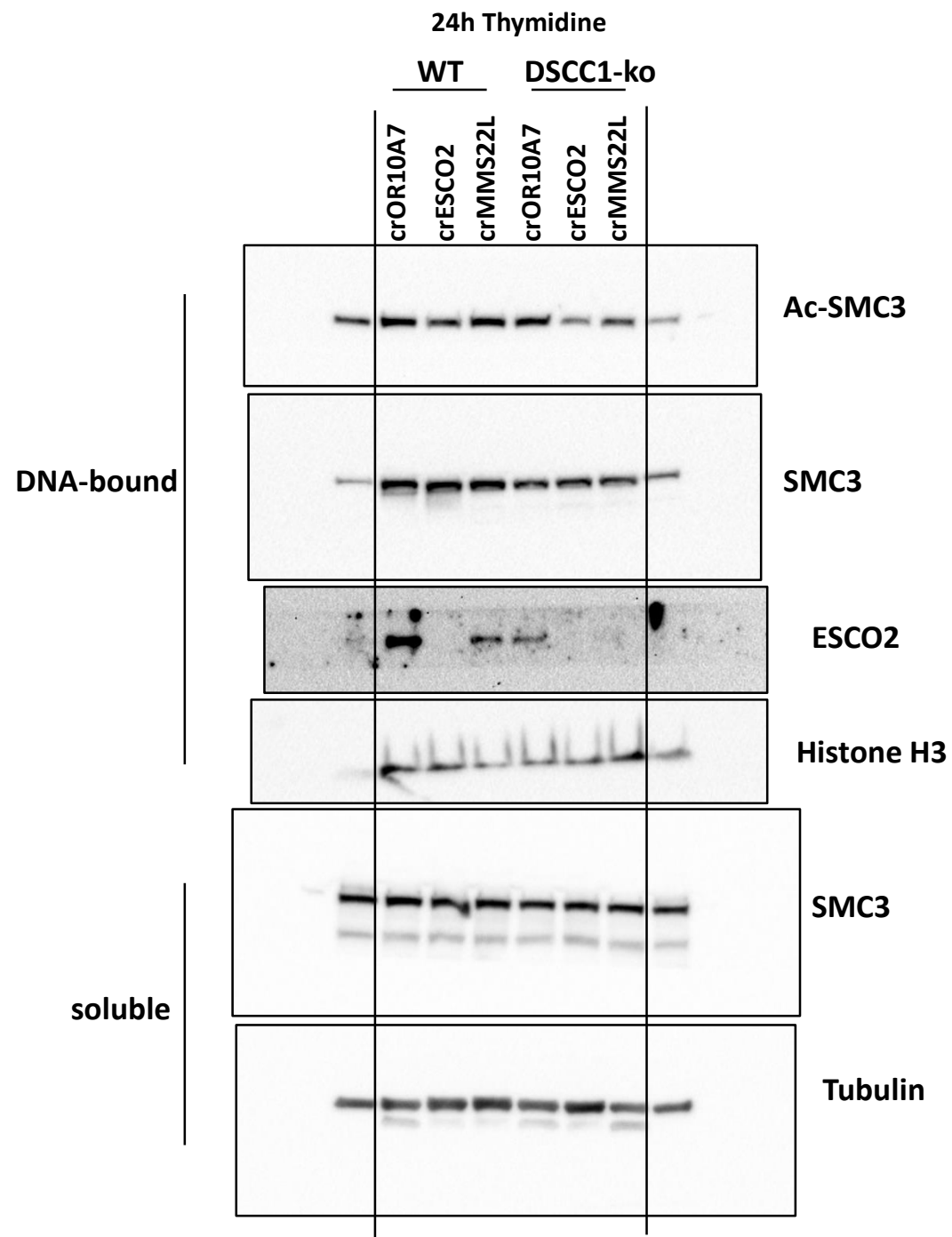

Supplement: Supplementary file 6 [file LSA-2022-01596_SdataF4.2.pdf]
